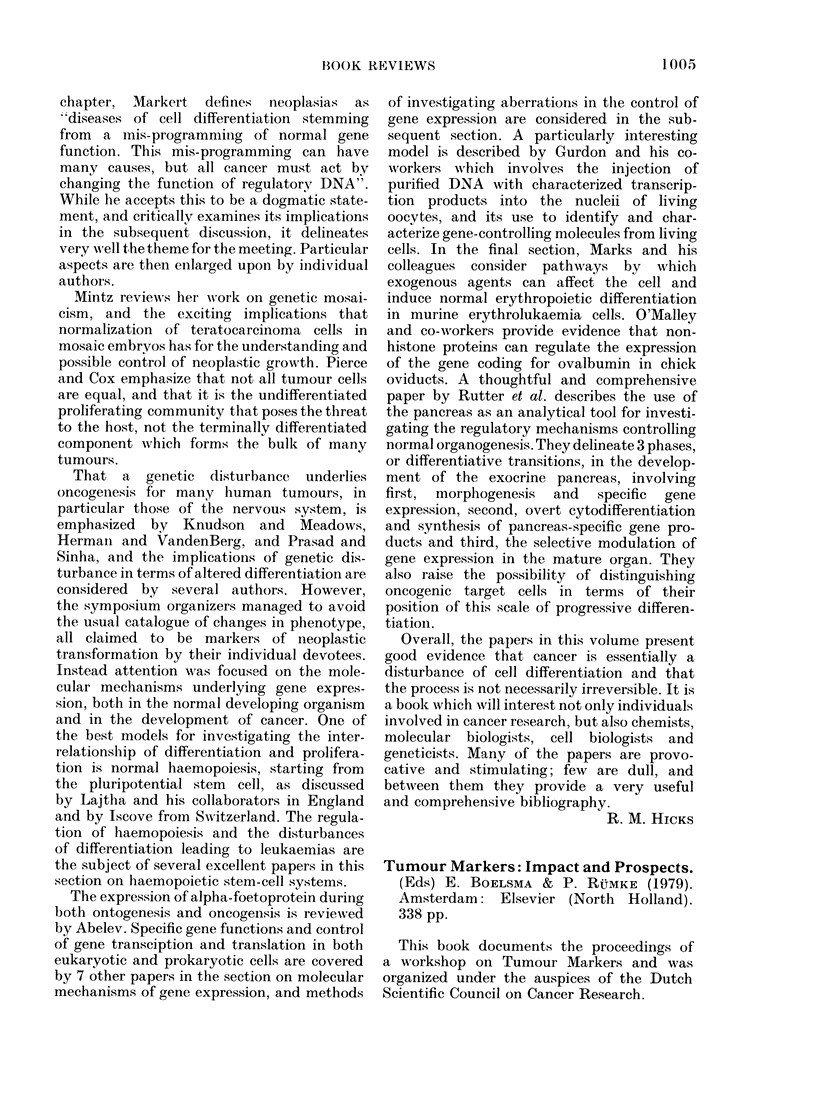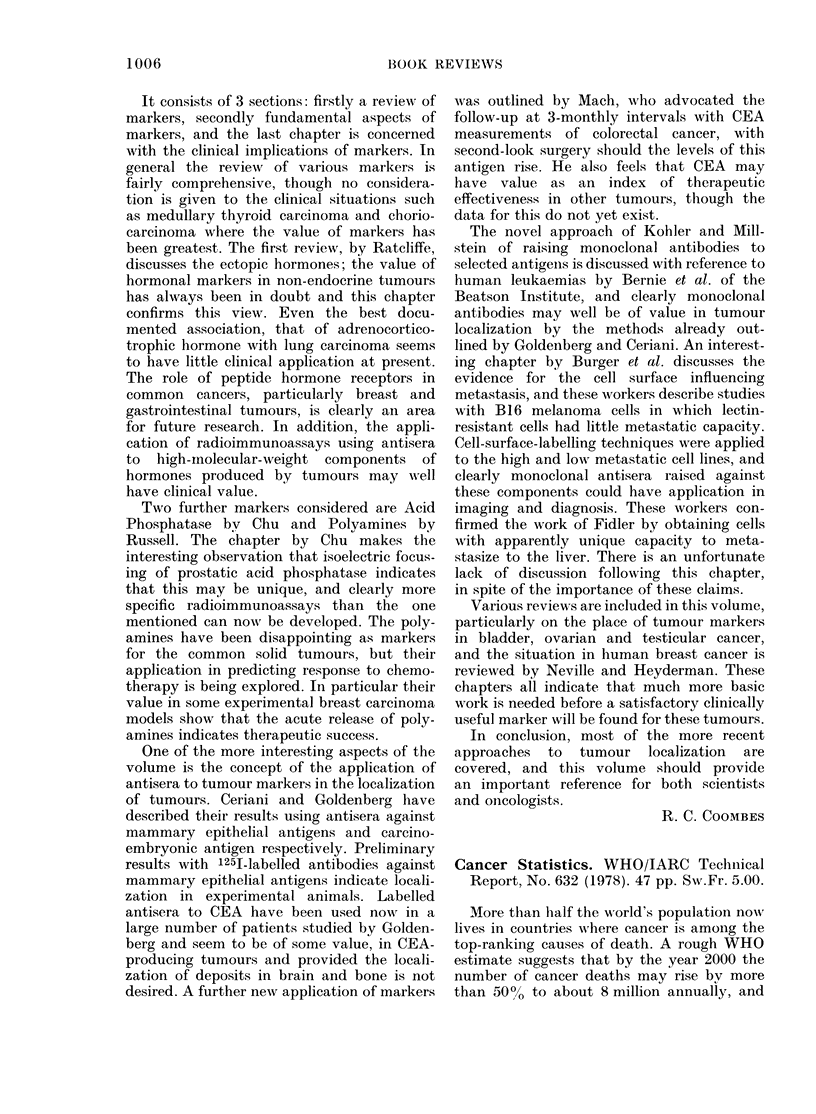# Tumour Markers: Impact and Prospects

**Published:** 1980-06

**Authors:** R. C. Coombes


**Tumour Markers: Impact and Prospects.** (Eds) E. Boelsma & P. Rümke (1979). Amsterdam: Elsevier (North Holland). 338 pp.

This book documents the proceedings of a workshop on Tumour Markers and was organized under the auspices of the Dutch Scientific Council on Cancer Research.

It consists of 3 sections: firstly a review of markers, secondly fundamental aspects of markers, and the last chapter is concerned with the clinical implications of markers. In general the review of various markers is fairly comprehensive, though no consideration is given to the clinical situations such as medullary thyroid carcinoma and choriocarcinoma where the value of markers has been greatest. The first review, by Ratcliffe, discusses the ectopic hormones; the value of hormonal markers in non-endocrine tumours has always been in doubt and this chapter confirms this view. Even the best documented association, that of adrenocorticotrophic hormone with lung carcinoma seems to have little clinical application at present. The role of peptide hormone receptors in common cancers, particularly breast and gastrointestinal tumours, is clearly an area for future research. In addition, the application of radioimmunoassays using antisera to high-molecular-weight components of hormones produced by tumours may well have clinical value.

Two further markers considered are Acid Phosphatase by Chu and Polyamines by Russell. The chapter by Chu makes the interesting observation that isoelectric focusing of prostatic acid phosphatase indicates that this may be unique, and clearly more specific radioimmunoassays than the one mentioned can now be developed. The polyamines have been disappointing as markers for the common solid tumours, but their application in predicting response to chemotherapy is being explored. In particular their value in some experimental breast carcinoma models show that the acute release of polyamines indicates therapeutic success.

One of the more interesting aspects of the volume is the concept of the application of antisera to tumour markers in the localization of tumours. Ceriani and Goldenberg have described their results using antisera against mammary epithelial antigens and carcinoembryonic antigen respectively. Preliminary results with $^{125}$I-labelled antibodies against mammary epithelial antigens indicate localization in experimental animals. Labelled antisera to CEA have been used now in a large number of patients studied by Goldenberg and seem to be of some value, in CEA-producing tumours and provided the localization of deposits in brain and bone is not desired. A further new application of markers was outlined by Mach, who advocated the follow-up at 3-monthly intervals with CEA measurements of colorectal cancer, with second-look surgery should the levels of this antigen rise. He also feels that CEA may have value as an index of therapeutic effectiveness in other tumours, though the data for this do not yet exist.

The novel approach of Kohler and Millstein of raising monoclonal antibodies to selected antigens is discussed with reference to human leukaemias by Bernie *et al.* of the Beatson Institute, and clearly monoclonal antibodies may well be of value in tumour localization by the methods already outlined by Goldenberg and Ceriani. An interesting chapter by Burger *et al.* discusses the evidence for the cell surface influencing metastasis, and these workers describe studies with B16 melanoma cells in which lectin-resistant cells had little metastatic capacity. Cell-surface-labelling techniques were applied to the high and low metastatic cell lines, and clearly monoclonal antisera raised against these components could have application in imaging and diagnosis. These workers confirmed the work of Fidler by obtaining cells with apparently unique capacity to metastasize to the liver. There is an unfortunate lack of discussion following this chapter, in spite of the importance of these claims.

Various reviews are included in this volume, particularly on the place of tumour markers in bladder, ovarian and testicular cancer, and the situation in human breast cancer is reviewed by Neville and Heyderman. These chapters all indicate that much more basic work is needed before a satisfactory clinically useful marker will be found for these tumours.

In conclusion, most of the more recent approaches to tumour localization are covered, and this volume should provide an important reference for both scientists and oncologists.

R. C. COOMBES